# Supplementary material for: New Evaluation of Isoflavone Exposure in the French Population
Source: Nutrients. 2019 Sep 28;11(10):2308. doi: 10.3390/nu11102308 (PMC6835759; doi:10.3390/nu11102308)
Supplement: Supplementary file 1 [file nutrients-11-02308-s001.pdf]

New evaluation of isoflavone exposure in the French population

Alexandre Lee[1], Laetitia Beaubernard[1], Valérie Lamothe[1] and Catherine Bennetau-Pelissero*[2].

1. Bordeaux Sciences Agro, F-33175 Gradignan, France
2. University of Bordeaux, Pharmacy Faculty, F-33077 Bordeaux, France

Abstract The study relates the present evaluation of exposure to estrogenic isoflavones of French consumers through two approaches: (1) identification of the isoflavone sources in the French food offering, (2) a consumption-survey on premenopausal women. For the foodstuff approach 150 food-items were analysed for genistein and daidzein. 12,707 labels of processed-foods from French supermarket websites and a restaurant-supplier website were screened. 1,616 foodstuffs of interest were retained. Phytoestrogens exposure was considered via soy, pea, broad bean and lupine. A price analysis was performed. 270 premenopausal women from the French metropolitan territory were interviewed for their global diet habits and soy consumption and perception. In supermarkets, there were significantly less selected foodstuffs containing soy than in restaurant (11.76% *vs* 25.71% $p<0.01$). There was significantly more soy in low price-foodstuff in supermarket ($p<0.01$). Isoflavone levels ranged from 81 to 123,871 μg per portion of the analyzed soy containing foodstuff. Among the women inquired 46.3% claimed having soy regularly. Isoflavone intake >45mg/day is associated to vegan-diet ($p<0.01$). In total, 11.9% of soy-consumers had a calculated isoflavone intake >50mg/day. This dose can lengthen the menstrual cycles. The actual exposure to phytoestrogen is likely to have an effect in a part of the French population.

*Corresponding author:
Catherine Bennetau-Pelissero
University of Bordeaux
Pharmacy Faculty
146 rue Léo Saignat, 33 077 Bordeaux, FRANCE
catherine.bennetau@u-bordeaux.fr
00 33 (0) 557 571 010 ext 1271

Running Title: Estrogenic isoflavone exposure in France

Keywords: Exposure, Foodstuff labelling, Legumes, Isoflavones, Phytoestrogens, Premenopausal women, Health, Endocrine disruptors

Summary

Page

Table S1. Isoflavone content in legumes found in processed food according to the scientific literature and references ………………………………………………………………………………………………….. 3

Table S2. Number of products screened in each survey performed in supermarkets ….…………………. 5

Table S3. Socio-demographic characteristics of the population interviewed in the study ……………..…. 6

Table S4. Margin of error associated with the observed proportions (for 270 respondents, with a confidence interval of 95%) …………………….…………………………………………………………………………6

Table S5. Genistein and Daidzein measurements in French foodstuff …………………….……..……… 7

Table S6. Price-quintiles for the analysis of the supermarket offering ………………...………..………..12

Table S7. Theoretical isoflavone content in canned legumes …………………………………....………..13

Table S8. Number of products indexed from the restaurant supplier web-site and containing legumes or suspect ……………………………………………………………………………….…..………………14

Table S9. Isoflavone concentrations in different soy-juice batches from different trade-marks ……..….. 14

Table S10: Data considered for the calculation of the isoflavone exposure ………………………………...15

Table S10a. Data considered for the calculation of the isoflavone exposure via soy-based food. Coefficients reflect the claimed frequency of consumptions ……….…………………………………….…….…..….15

Table S10b. Data considered for the calculation of the isoflavone exposure. Coefficients reflect the claimed frequency of consumptions …………………………………………………………………….…. 15

Figure S1. Number of products by categories found on supermarket website and their content in soy, pea, plant protein as well as those being suspect. ………………………………..……………………………... 16

Figure S2. Characterisation of the soy-based foodstuffs offer 17

Figure S3. Proportions of each type of canned legumes in the offer of one French retailer ……………... 17

Supplementary data S1: Survey questionnaire ………………..…….……………………….…………..... 18

Table S1. Isoflavone content in legumes found in processed food according to the scientific literature

Legumes	Biochanin A (CAS 491-80-5)	Daidzein (CAS 486-66-8)	Formononetin (CAS 485-72-3)	Genistein (CAS 446-72-0)	Total µg/100g	References
			µg/100g of fresh weight			
Fava (Raw)	na	330	na	150	**760**	USDA database, 2008
Fava (Raw)	na	na	500	1 990	**2 490**	Kaufman *et al.*, 1997
Fava (Raw)	na	7	na	6	**13**	Liggins *et al.*, 2002
Fava (Raw)	na	24	na	nd	**24**	Mazur *et al.*, 1996
**Mean Fava Raw**					**822**	
Lupine	na	10	na	1 036	**1 046**	Katagiri *et al.*, 2000
Lupine seed (*L. albus*)	na	4 660	na	5 920	**10 580**	Kaufman *et al.*, 1997
Lupine seed (*L. albus*)	na	1 900	na	6 520	**8 420**	Kaufman *et al.*, 1997
Lupine seed (*L. luteus*)	na	10	na	6 150	**6 160**	Kaufman *et al.*, 1997
Lupine seed (*L. albus*)	na	na	na	419	**419**	Sirtori *et al.*, 2004
Lupine seed (*L. mutabilis*)	na	na	na	61 500	**61 500**	Galvez-Ranilla *et al.*, 2009
**Mean Lupine seed**		**1645**		**13 591**	**14 688**	
Peas (*Pisum sativum*)*	na	52,9	na	49,7	**103**	de Kleijn *et al.*, 2001
Peas (*Pisum sativum*)*	na	268,8	na	69,8	**339**	Timoracká *et al.*, 2010
Peas raw (*Pisum sativum*)	na	220	na	0,0	**220**	Dvořák *et al.*, 2005
Yellow Split Peas (*P sativum*)	86	nd	nd	nd	**86**	Franke *et al.*, 1995
Green Split Peas (*P sativum*)	nd	726	nd	nd	**726**	Franke *et al.*, 1995
**Mean Peas**					**2 517**	
Soy Flour	na	94 969,26	na	142 167,63	**237 136,89**	Eldridge, 1982
Soy Flakes	na	41 955,43	na	140 960,01	**182 915,44**	Seo and Morr, 1984
Soy Flakes	na	72 038,90	na	122 111,68	**194 150,59**	Eldridge and Kwoleck 1983
Soy protein isolate 1	na	6 060,63	na	19 487,65	**25 548,3**	Mantovani *et al.*, 2009
Soy protein isolate 2	na	2 684,68	na	7 282,41	**9 967,1**	Mantovani *et al.*, 2009
Soy bean	30	47 000,00	70	74 000,00	**121 100,0**	Rochfort *et al.*, 2007
**Mean soy products**					**128 470**	

* in µg for 100g of dry weight

References included in Table S1

de Kleijn, M.J.; van der Schouw, Y.T.; Wilson, P.W.; Adlercreutz, H.; Mazur, W.; Grobbee, D.E.; Jacques, P.F. Intake of dietary phytoestrogens is low in postmenopausal women in the United States: the Framingham study(1-4). *J. Nutr.* **2001**, *131(6)*, 1826-1832.

Dvořák, R.; Pechová, A.; Pavlata, L.; Filípek, J.; Dostálová, J.; Réblová, Z.; Klejdus, B.; Kovařčík, K.; Poul, J. Reduction in the content of antinutritional substances in pea seeds (*Pisum sativum* L.) by different treatments. *Czech J. Anim. Sci.* **2005**, *50(11)*, 519–527.

Eldridge, A.C. High performance liquid chromatography separation of soybean isoflavones and their glucosides. *J. Chromatography B*. **1982**, *234*, 494-496.

Eldridge, A.C.; Kwolek, W.F. Soybean isoflavones: effect of environment and variety on composition. *J Agric Food Chem*. **1983**, *31(2)*, 394-396.

Franke, A.A.; Custer, L.J.; Cerna, C.M.; Narala, K. Rapid HPLC analysis of dietary phytoestrogens from legumes and from human urine. *Proc. Soc. Exp. Biol. Med*. **1995**, *208(1)*, 18-26.

Gálvez Ranilla, L.; Genovese, M.I.; Lajolo, F.M. Isoflavones and antioxidant capacity of Peruvian and Brazilian lupin cultivars. *J. Food Comp. Anal*. **2009**, *22*, 397-404.

Katagiri, Y.; Ibrahim, R.K.; Tahara, S. HPLC analysis of white lupin isoflavonoids. *Biosci. Biotechnol. Biochem*. **2000**, *64(6)*, 1118-1125.

Kaufman, P.B.; Duke, J.A.; Brielmann, H.; Boik, J.; Hoyt, J.E. A comparative survey of leguminous plants as sources of the isoflavones, genistein and daidzein: implications for human nutrition and health. *J. Altern. Complement. Med*. **1997**, *3(1)*, 7-12.

Liggins, J.; Mulligan, A.; Runswick, S.; Bingham, S.A. Daidzein and genistein content of cereals. *Eur. J. Clin. Nutr*. **2002**, *56(10)*, 961-966.

Mantovani, D.; Cardozo Filho, L.; Santos, L.C.; de Souza, V.L.; Watanabe, C.S. Chromatographic quantification of isoflavone content from soy derivates using HPLC technique. *J. Chromatogr. Sci.* **2009**, *47(9)*, 766-769.

Mazur, W.M.; Duke, J.A.; Wähälä, K.; Rasku, S.; Adlercreutz, H.; Isoflavonoids and lignans in legumes: Nutritional and health aspects in humans *J. Nutr. Biochem*. **1998**, *9*, 193-200.

Rochfort, S.; Panozzo, J. Phytochemicals for Health, the Role of Pulses. *J Agric Food Chem*. **2007**, *55*, 7981-7994.

Seo, A.; Morr, C.V. Improved high-performance liquid chromatographic analysis of phenolic acids and isoflavonoids from soybean protein products. *J Agric Food Chem*. **1984**, *32(3)*, 530–533.

Sirtori, C.R.; Lovati, M.R.; Manzoni, C.; Castiglioni, S.; Duranti, M.; Magni, C.; Morandi, S.; D'Agostina, A.; Arnoldi, A. Proteins of White Lupine Seed, a Naturally Isoflavone-Poor Legume, Reduce Cholesterolemia in Rats and Increase LDL Receptor Activity in HepG2 Cells. *J Nutr*. **2004**, *134*, 18-23.

Timoracká, M.; Vollmannová, A. Determination of flavonoids content in colored peas (*Pisum Sativum* L.) in relation to cultivar's dependence and storage duration under natural conditions. *Potravinarstvo* **2010**, *4(3)*, 58-62.

Table S2. Number of products screened in each survey performed in supermarkets

Category of analyzed products	Number of products per category			
	Total product screened per category*	Global survey of food claiming containing legumes	Supplier survey for price analysis	Survey for percentage analysis of food claiming containing legumes
Baby-food	651	0	0	0
Biscuits	699	44	0	0
Bread	155	52	50	52
Breaded or battered fish and minced fish portion	25	0	0	17
Breaded and battered meat	32	29	29	29
Canned legumes	307	54	54	0
Cooking aids and sauces	201	32	32	32
Dairy products (except-soy products)	2146	2	0	0
Delicatessen	635	26	26	49
Battered meat and Nuggets	85	23	23	21
Gluten free foodstuffs	98	27	78	78
Ice-cream and desert	123	16	16	16
Minced meat fresh or frozen	154	65	65	22
Pizza	452	2	0	0
Processed canned food	408	26	26	26
Processed dishes	145	57	57	57
Snacks or Surimi	102	32	31	75
Soy-based products	257	138	0	0
**Total per inquiry method**	**6675**	**625**	**487**	**474**
**Total products indexed from supermarket**			**1112**	

* overview of 5 main suppliers in France

Table S3. Socio-demographic characteristics of the population interviewed in the study

Age		18-25	26-30	31-35	36-40	41-50
	Observed	106	51	29	25	59
	%	39.3%	18.9%	10.7%	9.3%	21.9%
	National*	22%	15%	15%	16%	33%
Number of persons at home		1	2	3	4	5 and over
	Observed	71	75	47	55	22
	%	26.3%	27.8%	17.4%	20.4%	8.1%
	National*	29%	23%	19%	19%	10%
Children or not		yes		no		
	Observed	95		175		
	%	35.2%		64.8%		
	National*	47%		53%		
Family monthly incomes		<1500€	[1500 - 2500 €[	[2500 - 4000 €[	[4000 - 6000 €[	>6000 €
	Observed	75	77	79	26	8
	%	28.3%	29.1%	29.8%	9.8%	3%
	National**	31%	44%	15%	5%	3%

According to the data "National" means ether Insee: * or French Ministry of Finance: **

* https://www.insee.fr/fr/statistiques/

** https://www.impots.gouv.fr/portail/statistiques

Table S4: Margin of error associated with the observed proportions (for 270 respondents, with a confidence interval of 95%)

Observed proportion	5 %	10 %	20 %	30 %	40 %	50 %	60 %	70 %	80 %	90 %	95 %
Margin of error	*2.6 %*	*3.6 %*	*4.8 %*	*5.5 %*	*5.8 %*	*6.0 %*	*5.8 %*	*5.5 %*	*4.8 %*	*3.6 %*	*2.6 %*
Lower boundary	*2.4 %*	*6.4 %*	*15.2 %*	*24.5 %*	*34.2 %*	*44 %*	*54.2 %*	*64.5 %*	*75.2 %*	*86.4 %*	*92.4 %*
Upper boundary	*7.6 %*	*13.6 %*	*24.8 %*	*35.5 %*	*45.8 %*	*56 %*	*65.8 %*	*75.5 %*	*84.8 %*	*93.6 %*	*97.6 %*

Table S5. Genistein and Daidzein measurements in French foodstuff
(all data are given in aglycone equivalent per wet weight)

		Genistein (µg/g)	Daidzein (µg/g)	Total (µg/g)	Reasonable portion size	Intake for 1 portion (µg)
**Foodstuffs based on soy-juice**						
1	Tonyu 1	91.37 (± 6.52)	49.57 (± 3.47)	140.94 (± 9.99)	1 bowl (350 mL)	49,330
2	Tonyu 2	51.32 (± 8.16)	39.83 (± 4.39)	91.14 (± 12.55)	1 bowl (350 mL)	31,899
3	Tonyu 3	53.21 (± 12.47)	37.33 (± 4.11)	90.54 (± 16.58)	1 bowl (350 mL)	31,690
4	Tonyu 4	32.84 (± 9.48)	19.24 (± 4.59)	52.08 (± 14.06)	1 bowl (350 mL)	18,232
5	Tonyu 5	60.80 (± 7.21)	44.82 (± 3.38)	105.62 (± 10.59)	1 bowl (350 mL)	36,971
6	Tonyu 6	29.96 (± 1.92)	16.91 (± 1.94)	46.88 (± 3.89)	1 bowl (350 mL)	16,413
7	Tonyu 7	15.68 (± 3.60)	12.64 (± 2.38)	28.33 (± 5.97)	1 bowl (350 mL)	9,914
8	Soy-juice chocolate taste 1	104.54 (± 12.76)	52.67 (± 7.55)	157.21 (± 20.31)	1 mug (250 mL)	39,302
9	Soy-juice chocolate taste 2	107.00 (± 7.49)	70.00 (± 4.92)	178.75	1 mug (250 mL)	44,687
10	Soy-based Yoghurts 1	44.70 (± 3.17)	37.40 (± 3.61)	82.20 (± 6.78)	1 yoghurt (100 g)	8,220
11	Soy-based Yoghurts 2	33.17 (±8.47)	7.78 (± 1.00)	40.95 (± 9.47)	1 yoghurt (100 g)	4,104
12	Soy-based Yoghurts 3	76.51 (±17.72)	48.68 (± 8.64)	125.19 (± 26.37)	1 yoghurt (125 g)	15,652
13	Soft soy-based yoghurt	125.00 (± 8.75)	129.19 (± 9.12)	254.31 (± 17.87)	1 yoghurt (125 g)	30,510
14	Natural soy yogurt	64.23 (± 14.18)	35.33 (± 6.22)	99.56 (±20.40)	1 yoghurt (100 g)	9,955
15	Soy-based yoghurt with peach	106.71 (± 4.78)	58.69 (± 10.33)	165.39 (±15.10)	1 yoghurt (100 g)	16,539
16	Soy-based Chocolate cream 1	52.41 (± 5.63)	28.82 (± 5.07)	81.23 (±10.71)	1 yoghurt (125 g)	10,153
17	Soy-based Chocolate cream 2	67.13 (± 13.96)	38.63 (± 4.52)	105.76 (± 18.48)	1 cup (100 g)	10,582
18	Soy-based Chocolate cream 3	26.26 (± 3.62)	14.12 (± 2.66)	40.39 (± 6.28)	1 cup (100 g)	4,044
19	Soy-based Chocolate cream 4	40.94 (± 8.87)	19.65 (± 2.16)	60.59 (± 11.02)	1 cup (100 g)	6,067
20	Soy-based Chocolate cream 5	13.95 (± 3.56)	3.34 (± 0.47)	17.29 (± 4.03)	1 cup (100 g)	1,733
21	Soy-based Chocolate cream 6	20.25 (± 2.07)	14.91 (± 3.59)	35.16 (± 5.66)	1 cup (100 g)	3,521
22	Soy-based Chocolate cream 7	34.58 (±6.69)	23.24 (± 3.68)	57.81 (± 10.36)	1 cup (100 g)	5,782
23	Soy-based Vanilla soy cream 1	29.82 (± 2.16)	19.39 (± 1.36)	49.21 (± 2.87)	1 cup (100 g)	4,921

Table S5 Continued

		Genistein (µg/g)	Daidzein (µg/g)	Total (µg/g)	Reasonable portion size	Intake for 1 portion (µg)
24	Soy-based Vanilla soy cream 2	110.556 (± 28.58)	60.80 (± 10.70)	171.35 (± 39.28)	1 cup (125 g)	21,418
25	Soy-based Caramel soy cream	40.00 (± 2.86)	17.90 (± 0.7)	57.89 (± 2.87)	1 cup (100 g)	5,789
26	Soy-based Vanilla dessert	159.50 (± 11.16)	63.20 (± 4.42)	224.10 (± 2.87)	1 cup (100 g)	22,410
27	Herb cheese made of soy 1	368.39 (± 25.86)	357.23 (± 22.99)	725.62 (± 58.85)	1 portion (50 g)	36,280
28	Herb cheese made of soy 2	351.12 (± 22.36)	300.93 (± 20.94)	654.31 (± 43.30)	1 portion (50 g)	32,710
29	Herb cheese made of soy 3	285.80 (± 15.26)	185.15 (± 12.34)	470.95 (± 27.60)	1 portion (50 g)	23,640
30	Nature cheese made of soy	43.42 (± 9.02)	15.03 (± 2.91)	58.45 (±11.99)	1 portion (50 g)	2,923
31	Soy-based cream substitute 1	70.08 (± 5.11)	63.16 (± 4.42)	134.95 (± 9.53)	1 portion (50 mL)	6,750
32	Soy-based cream substitute 2	32.98 (± 0.69)	28.83 (± 4.85)	61.81 (± 5.54)	1 portion (50 mL)	3,090
33	Soy-based cream substitute 3	56.04 (± 5.13)	34.42 (± 2.21)	90.46 (± 7.34)	1 portion (50 mL)	4,522
34	Soy-based cream substitute 4	50.69 (± 5.29)	27.49 (± 1.92)	78.1 (± 7.21)	1 portion (50 mL)	3,913
35	Soy-based cream substitute 5	54.27 (± 17.57)	29.84 (± 5.25)	54.27 (± 17.57)	1 portion (50 mL)	2,713
**Asian types soy-based dishes**						
36	Smoked tempeh	165.33 (± 11.56)	112.00 (± 7.84)	277.33 (± 19.40)	3 slices (50 g)	13,870
37	Japanese soft Tofu	117.87 (± 10.29)	70.92 (± 2.88)	188.79 (± 13.18)	1 portion (100 g)	18,879
38	Soft Tofu	77.60 (± 10.29)	42.81 (± 3.63)	120.41 (± 16.56)	1 portion (100 g)	12,041
39	Natural Tofu 1	225.27 (± 75.14)	117.22 (± 7.43)	342.49 (± 82.57)	1 portion (125 g)	42,810
40	Natural Tofu 2	119.43 (± 35.08)	101.11 (± 24.11)	220.54 (± 59.19)	1 portion (100 g)	22,054
41	Traditional Tofu*	224.43 (± 11.79)	100.92 (± 12.48)	325.35 (± 24.26)	1 portion (100 g)	32,535
42	Whey from Traditional Tofu*	744.36 (± 39.89)	459.93 (± 60.17)	1.204.30 (± 100.06)	in 1 pack (60 mL)	72,258
43	Asian Tofu	48.00 (± 3.26)	46.16 (± 3.16)	95.27 (± 6.42)	1 portion (100 g)	9,530
44	Breaded Tofu 1	150.33 (± 10.52)	71.22 (± 5.57)	221.55 (± 16.09)	1 portion (100 g)	22,150
45	Breaded Tofu 2	289.29 (± 6.04)	188.49 (± 2.00)	477.78 (± 8.04)	1 portion (100 g)	47,778
46	Smoked Tofu	273.54 (± 15.33)	178.54 (± 5.50)	452.08 (± 20.83)	1 portion (100 g)	45,210
47	Tofu with garlic	216.74 (± 9.60)	138.25 (± 4.09)	354.99 (± 13.69)	1 portion (80 g)	28,400
48	Tofu with curry	411.96 (± 105.56)	226.58 (± 39.87)	638.54 (± 145.44)	1 portion (125 g)	79,817
49	Soy sauce 1	5.64 (± 1.55)	10.36 (± 2.63)	16.00 (± 4.18)	1 portion (10 mL)	160

Table S5 Continued

		Genistein (μg/g)	Daidzein (μg/g)	Total (μg/g)	Reasonable portion size	Intake for 1 portion (μg)
**Prepared dishes based on soy**						
50	Soy sauce 2	5.55 (± 1.62)	11.27 (± 2.87)	16.82 (± 4.49)	1 portion (10 mL)	162
51	Sausages made of soy 1	82.21 (± 5.76)	40.64 (± 2.87)	122.85 (± 8.63)	2 sausages (90 g)	11,060
52	Sausages made of soy 2	134.15 (± 10.55)	66.95 (± 5.53)	201.10 (± 16.08)	3 sausages (80 g)	16,090
53	Sausages made of soy 3	259.50 (± 10.42)	231.00 (± 20.70)	490.50 (± 31.12)	2 sausages (90 g)	44,145
54	Sausages made of soy 4	132.05 (± 16.89)	42.74 (± 6.85)	174.79 (±23.74)	3 sausages (160 g)	27,970
55	Soy biscuits with figs	95.38 (± 6.36)	87.74 (± 6.16)	183.12 (± 12.52)	4 biscuits (80 g)	14,650
56	Buckweat pancakes with tofu	228.50 (± 15.86)	154.00 (± 10.35)	382.50 (± 26.21)	1 pancake (100 g)	38,250
57	Soy pancakes with tomatoes	202.30 (± 14.16)	116.92 (± 8.56)	319.22 (± 22.72)	1 pancake (100 g)	31,920
58	Soy pancakes "provençale"	227.15 (± 15.57)	129.48 (± 11.02)	356.63 (± 26.59)	1 pancake (100 g)	35,663
59	Legumes with tonyu sauce	98.81 (± 1.90)	62.90 (± 6.35)	161.71 (± 8.25)	1 dish (300 g)	48,510
60	Vegan Rice with soy & vegetables	132.29 (± 12.18)	72.76 (± 12.81)	205.04 (± 24.97)	1 dish (250 g)	51,261
61	Vegan Steak Tomato & onions	332.44 (± 11.39)	222.65 (± 3.29)	555.09 (± 14.68)	1 steak (90 g)	49,960
62	Vegan Steak Tomato & onions	288.62 (± 3.69)	234.93 (± 51.66)	523.55 (± 55.36)	1 steak (90 g)	47,119
63	Vegan Steak with vegetable	122.55 (± 10.29)	118.92 (± 2.64)	241.47 (± 12.93)	1 steak (100 g)	24,146
64	Soy Steak Tomato & cereal	190.42 (± 7.12)	133.53 (± 10.40)	323.96 (± 17.52)	1 steak (90 g)	29,156
65	Croq Tofou « provençale »	165.42 (± 5.00)	112.18 (± 8.94)	277.61 (± 13.94)	1 steak (100 g)	27,760
66	Vegan Steak tomato & herbs	164.62 (± 10.02)	119.38 (± 6.31)	284.00 (± 16.33)	1 steak (100 g)	28,400
67	Vegan Steak with pepper	147.50 (± 9.23)	103.63 (± 11.74)	251.13 (± 20.96)	1 steak (80 g)	20,090
68	Soy Steak and vegetables	20.37 (± 2.31)	29.35 (± 10.49)	49.73 (± 12.80)	1 steak (100 g)	4,970
69	Indian Soy Steak with Curry	123.10 (± 13.76)	91.93 (± 4.02)	215.03 (± 17.78)	1 steak (100 g)	21,500
70	Soy Steak tomato & herbs	24.07 (± 4.63)	17.75 (± 2.86)	41.83 (±7.49)	1 steak (100 g)	4,180
71	Breaded Soy Steak	159.52 (± 38.26)	87.73 (± 15.44)	247.25 (± 53.70)	1 steak (90 g)	22,252
72	Vegan steak	174.67 (±46.38)	96.07 (±16.91)	270.74 (±63.28)	1 steak (100 g)	27,070
73	Vegan Grill	53.53 (± 6.04)	29.44 (± 5.18)	82.97 (± 11.23)	1 steak (90 g)	7,467
74	Vegan escalope grilled	45.81 (± 14.21)	25.19 (± 4.43)	71.01 (± 18.64)	1 steak (80 g)	5,680
75	Soy Square	203.92 (± 37.28)	112.16 (± 19.74)	316.08 (± 57.02)	1 steak (100 g)	31,608
76	Vegan "Bolognaise for pasta" 1	244.73 (± 81.73)	155.40 (± 8.25)	400.13 (± 89.98)	1 portion (120 g)	48,020
77	Vegan "Bolognaise for pasta" 2	127.28 (± 21.29)	94.88 (± 21.29)	222.16 (± 21.98)	1 portion (125 g)	27,770

Table 5 (continued)

		Genistein (µg/g)	Daidzein (µg/g)	Total (µg/g)	Reasonable portion size	Intake for 1 portion (µg)
78	Vegan chopped dish for pasta 3	70.93 (± 6.46)	62.45 (± 3.66)	133.38 (± 10.12)	1 portion (150 g)	20,012
79	Cuscus vegan	164.61 (± 18.44)	116.89 (± 6.05)	281.51 (± 24.50)	1 portion (250 g)	70,382
80	Soy. rice and vegetables	48.14 (± 6.08)	31.95 (± 4.13)	80.09 (± 10.21)	1 portion (250 g)	20,023
81	Vegan nuggets 1	21.10 (± 3.40)	7.78 (± 1.26)	28.88 (± 4.65)	1 portion (150 g)	4,333
82	Vegan nuggets 2	20.36 (± 2.42)	10.09 (± 1.83)	30.45 (± 4.25)	1 portion (100 g)	3,044
83	Vegan croq' with cheese	43.85 (± 8.77)	14.63 (± 0.65)	58.48 (± 9.42)	1 portion (100 g)	5,852
84	Vegan patty	100.07 (± 16.36)	79.37 (± 3.41)	179.44 (±19.78)	1 portion (150 g)	26,924
**Soy products**						
85	Soy bean (fresh frozen)	260.28 (±29.85)	143.16 (±25.20)	403.44 (±55.04)	1 portion (200 g)	80,688
86	Soy flour	644.72 (±141.89)	354.60 (±62.41)	999.32 (±204.30)	1 portion (33 g)	32,978
87	Conventional soy grain	490.98 (± 6.62)	347.68 (± 78.26)	838.67 (± 84.88)	1 portion (100 g)	85,421
88	Toasted soy grain (appetisers) 1	1.360.00 (± 51.32)	1117.42 (± 119.03)	2.477.42 (± 170.62)	1 portion (50 g)	123,871
89	Toasted soy grain (appetisers) 2	370.11 (±45.14)	249.85 (±14.00)	619.97 (±59.14)	1 portion (50 g)	31,002
90	Toasted soy grain (appetisers) 3	616.00 (±89.08)	572.00 (±93.55)	1188.00 (±182.64)	1 portion (50 g)	59,404
91	Toasted soy grain (appetisers) 4	809.33 (±49.37)	666.67 (±102.63)	1476.00 (±152.00)	1 portion (50 g)	73,805
92	Toasted soy grain (appetisers) 5	650.67 (±68.86)	286.22 (±66.26)	936.89 (±135.12)	1 portion (50 g)	46,847
**Health products based on soy**						
93	Instant powder for drinks	99.69 (± 6.96)	106.11 (± 7.35)	205.80 (± 14.31)	3 spoons (25 g)	8,026
94	Powdered soy "milk"	1.310.00 (± 81.62)	1.070.00 (± 64.96)	2.390.00 (± 146.58)	3 doses (30 g)	43,974
95	Slimming dish (soup)	223.59 (± 15.46)	135.44 (± 9.24)	359.03 (± 24.70)	1 pack (50 g)	16,510
96	Slimming dish (breakfast)	185.03 (± 11.95)	98.36 (± 7.56)	283.39 (± 19.51)	1 pack (50 g)	13,030
97	Slimming dish (meal)	287.17 (± 18.53)	193.64 (± 13.22)	480.81 (± 31.75)	1 pack (50 g)	22,110
98	Cereal max energy	252.24 (± 12.75)	138.73 (± 24.42)	390.97 (± 37.16)	1 bar (165 g)	64,509
99	High protein 90	155.77 (± 10.52)	85.67 (± 15.08)	241.44 (± 25.60)	1 portion (100 g)	24,144
100	Soy lecithin Extract 1	0.17 (± 0.01)	0.68 (± 0.04)	0.97 (± 0.05)	1 portion (10 g)	9.7
101	Soy lecithin Extract 2	0.86 (± 0.06)	2.36 (± 0.16)	3.52 (± 0.22)	1 portion (10 g)	35.2
102	Whole weat loaf	1.76 (± 0.37)	2.51 (± 0.08)	4.27 (± 0.45)	1 portion (80 g)	340
103	Gluten free baguette bread	10.29 (± 1.93)	5.48 (± 1.10)	15.77 (± 3.03)	1 portion (80 g)	126
104	Organic Waffle with honey	5.91 (± 1.70)	13.37 (± 0.78)	19.28 (± 2.47)	1 portion (80 g)	154
105	"Bolognaise" Pasta	34.69 (± 4.96)	19.08 (± 3.36)	53.77 (± 8.32)	1 portion (360 g)	19,357

Table 5 (Continued)

		Genistein (µg/g)	Daidzein (µg/g)	Total (µg/g)	Reasonable portion size	Intake for 1 portion (µg)
106	Asian Rice	7.62 (± 3.81)	4.19 (± 0.74)	11.80 (± 4.55)	1 portion (220 g)	2,597
107	Small Ravioli	6.39 (± 1.14)	3.52 (± 0.62)	9.91 (± 1.76)	1 portion (250 g)	2,476
108	Minced beef pie (Parmentier)	4.66 (± 0.32)	1.53 (± 0.11)	6.20 (± 0.43)	1 portion (300 g)	1,860
109	Minced beef portions	73.92 (± 5.11)	49.34 (± 3.43)	122.26 (± 8.54)	1 steak (100 g)	12,226
110	Minced beef portions with onions	4.60 (± 0.44)	3.87 (± 0.67)	8.47 (± 1.12)	1 portion (100 g)	846
111	Stuffed tomatoes 1	33.02 (± 2.82)	26.94 (± 1.96)	59.99 (± 4.78)	2 tomatoes	8,960
112	Stuffed tomatoes 2	44.39 (± 1.39)	23.33 (± 1.41)	67.71 (± 2.80)	2 tomatoes	6,771
113	Stuffed cabbages	33.04 (± 2.55)	25.48 (± 1.53)	58.48 (± 4.08)	2 cabbages	9,040
114	Chicken sausages appetizers	4.97 (± 0.99)	3.22 (± 0.57)	8.19 (± 1.56)	1 portion (110 g)	900
**Foodstuffs with hidden soy**						
115	Chicken sausages	1.63 (± 0.35)	0.72 (± 0.05)	2.35 (± 0.40)	1 portion (100 g)	235
116	Frozen chicken nuggets	0.93 (± 0.25)	0.25 (± 0.12)	1.18 (± 0.37)	1 portion (120 g)	142
117	Chicken nuggets	11.93 (± 2.85)	5.39 (± 1.31)	17.32 (± 4.16)	1 portion (130 g)	2,251
118	Chicken nuggets	0.42 (± 0.12)	0.58 (± 0.02)	1.01 (± 0.13)	1 portion (100 g)	101
119	Small Beaf balls	54.17 (± 9.75)	18.99 (± 0.60)	73.17 (± 10.35)	1 portion (150 g)	10,970
120	Raw Beaf balls	0.19 (± 0.04)	0.35 (± 0.07)	0.54 (± 0.11)	1 portion (150 g)	81
121	Genuine Beaf Balls	38.24 (± 3.34)	23.08 (± 0.09)	61.32 (± 3.42)	1 portion (150 g)	9,198
122	Mini Beaf Balls	24.80 (± 1.61)	18.03 (± 5.23)	42.83 (± 6.83)	1 portion (150 g)	6,424
123	Bolo Balls	25.72 (± 1.75)	10.75 (± 1.60)	36.47 (± 3.35)	1 portion (130 g)	4,741
124	Meatballs (1)	78.14 (± 6.16)	54.23 (± 3.56)	132.37 (± 9.72)	4 balls (125 g)	16,546
125	Meatballs (2)	82.55 (± 6.68)	59.60 (± 2.59)	142.15 (± 9.27)	4 balls (125 g)	17,768
126	Minced veal (breaded)	55.96 (± 3.86)	35.32 (± 2.45)	91.28 (± 6.31)	1 steak (100 g)	9,128
127	Brownies	65.24 (± 4.56)	43.92 (± 3.15)	109.16 (± 7.71)	3 Pieces (90 g)	9,824

Figures are mean ± SD of 3 measures performed on 3 different microtitration plates

*The traditional tofu is an industrial product.

Table S6. Price-quintiles for the analysis of the supermarket offering

Price/kg	Breaded and battered Meat	Breaded and battered fish	Burgers	Delicatessen	Fish Steak	Meatballs	Meat flesh	Minced meat	Nuggets	Stuffed vegetables	Surimi
**Price 1**	<7.00€	<7.50€	<7.00€	<5.00€	12.00€ - 13.00€	<8.00€	<7.00€	<8.00€	<8.00€	<4.00€	<6.00€
**Price 2**	7.01€ - 8.00€	7.51€ - 9.50€	7.01€ - 11.00€	5.01€ - 8.00€	13.01€ - 14.00€	8.01€ - 10.00€	7.01€ - 9.00€	8.01€ - 11.00€	8.01€ - 10.00€	4.01€ - 5.50€	6.01€ - 9.00€
**Price 3**	8.01€ - 9.00€	9.51€ - 11.50€	11.01€ - 1.,00€	8.01€ - 11.00€	14.01€ - 15.00€	10.01€ - 12.00€	9.01€ - 11.00€	11.01€ - 14.00€	10.01€ - 12.00€	5.51€ - 7.00€	9.01€ - 12.00€
**Price 4**	9.01€ - 10.00€	11.51€ - 13.50€	15.01€ - 19.00€	11.01€ - 14.00€	15.01€ - 16.00€	12.01€ - 14.00€	11.01€ - 13.00€	14.01€ - 17.00€	12.01€ - 14.00€	7.01€ - 8.50€	12.01€ - 15.00€
**Price 5**	>10.00€	13.50€ - 15.50€	>19.00€	>14.00€	16.01€ - 17.00€	>14.00€	>13.00€	>17.00€	>14.01€	8.51€ - 10€	>15.00€

Table S7. Theoretical isoflavone content in canned legumes *(all data are given in aglycone equivalent per wet weight)*

	Mean theoretical isoflavone content µg/100g*						Mean % in canned products				
Legumes	Number of Items	Biochanin A	Daidzein	Formononetin	Genistein	Total	Peas	Beans	Flageolets	Lentils	References
Green flageolet beans	6	40	10	150	20	220		100			USDA Database, 2008
French beans	13	nd	nd	15	nd	15		100			Franke et al., 1995
Wax beans	1	18.5	nd	32.5	nd	51		100			Franke et al., 1995
White beans	2	30	nd	41	37	108		100			Franke et al., 1995
Lingot beans	1	1116	5,5	133.5	70	1325		100			Mazur et al., 1998
Red beans	2	79	3.33	nd	4.66	87		100			Horn-Ross et al., 2000
French and wax beans	1	7,4	nd	19	nd	26.4		40			Franke et al., 1995
Lentils	2	nd	8,5	nd	46,5	55				100	Ritchie et al., 2006
Mixed vegetables	3	8	716	33	258	1015	20	20	20		Dvořák et al., 2010
Mojette beans	1	1116	5,5	133.5	70	1325		100			Franke et al., 1995
Peas	6	na	3570	na	1270	4840	100				Dvořák et al., 2010
Peas & carrots mixed	5	na	2142	na	762	2904	60				Dvořák et al., 2010
Split beans	1	28.66	242	nd	nd	270.66		100			Franke et al., 1995
Chick peas	4	64	40	140	350	594	100				Franke et al., 1995
Mungo beans	1	1	566	9.33	1163	1739.33		100			Horn-Ross et al., 2000
Elaborated cans with meat	Mixed Cans	Biochanin A	Daidzein	Formononetin	Genistein	Total	Mean % in canned products				
Cassoulet (white beans)	2	9.9	nd	13.53	12.21	**35.64**		33			Franke et al., 1995
Cassoulet (lingot beans)	5	334.8	1.65	40.05	21	**397.5**		30			Mazur et al., 1998
Lentils & Sausages	1	nd	1.36	nd	7.44	**8.8**				16	Ritchie et al., 2006

Bhagwat, S.; Haytowitz, D.B.; Holden, J.M. USDA Database for the Isoflavone Content of Selected Foods., 2008, https://www.ars.usda.gov/ARSUserFiles/80400525/Data/isoflav/Isoflav_R2-1.pdf (Accessed July 2019)

Franke, A.A.; Custer, L.J.; Cerna, C.M.; Narala, K. Rapid HPLC analysis of dietary phytoestrogens from legumes and from human urine. *Proc Soc Exp Biol Med*. **1995**, *208(1)*, 18-26

Mazur, W.M.; Duke, J.A.; Wähälä, K.; Rasku, S.; Adlercreutz, H.; Isoflavonoids and lignans in legumes: Nutritional and health aspects in humans *J. Nutr. Biochem*, **1998(9)**, 193-200.

Horn-Ross, P.L.; Barnes, S.; Lee, M.; Coward, L.; Mandel, J.E.; Koo, J.; John, E.M.; Smith, M. Assessing phytoestrogen exposure in epidemiologic studies: development of a database (United States).*Cancer Causes Control*. **2000**, *11(4)*, 289-298.

Dvořák, R.; Pechová, A.; Pavlata, L.; Filípek, J.; Dostálová, J.; Réblová, Z.; Klejdus, B.; Kovařčík, K.; Poul, J. Reduction in the content of antinutritional substances in pea seeds (*Pisum sativum* L.) by different treatments. *Czech J Anim Sci*. **2005**, *50(11)*, 519–527.

Ritchie, M.R.; Cummings, J.H.; Morton, M.S.; Michael Steel, C.; Bolton-Smith, C.; Riches, A.C. A newly constructed and validated isoflavone database for the assessment of total genistein and daidzein intake. *Br J Nutr*. **2006,** *95(1)*, 204-213.

Table S8. Number of products indexed from the restaurant supplier web-site and containing legumes or suspect

Foodstuffs	Number of indexed products
1. Breaded or battered fish	109
2. Breaded or battered meat	28
3. Burgers	7
4. Delicatessen	139
5. Meat balls	16
6. Bulk minced-meat	5
7. Minced fish portions	1
8. Minced meat portions	69
9. Nuggets	14
10. Stuffed vegetables	22
11. Surimi	19
Total	429

Table S9: Isoflavone concentrations in different soy-juice batches from different French trade-marks *(all data are given in aglycone equivalent per wet weight)*

	Genistein µg/L	Daidzein µg/L	Total µg/L	For a mug 330 mL (µg)	For a mug 330 mL (mg)
Trade-mark 1	55,800	31,400	87,200	28,776	28.77
Trade-mark 2a	143,300	138,200	281,500	92,895	92.89
Trade-mark 2b	135,230	86,810	222,040	73,273	73.27
Trade-mark 2c	177,990	112,630	289,500	95,535	95.53
Trade-mark 2d	113,320	74,120	187,440	61,855	61.85
Trade-mark 2e	181,050	101,950	283,000	93,390	93.39
Trade-mark 3	82,700	46,300	129,000	42,570	42.57
Trade-mark 4a	78,300	63,700	142,000	46,860	46.86
Trade-mark 4b	51,320	39,920	91,240	30,109	30.11
Trade-mark 5	51,500	31,700	83,200	27,456	27.45
Trade-mark 6	32,840	19,240	52,080	17,186	17.17
Trade-mark 7	60,800	44,820	105,620	34,854	34.85
Trade-mark 8	29,960	16,910	46,880	15,470	15.47
Trade-mark 9	15,680	12,640	28,330	9,349	9.35
Trade-mark 10	104,540	52,670	157,210	47,163	47.16
Trade-mark 11	107,820	70,930	178,750	53,625	53.63

Table S10a. Data considered for the calculation of the isoflavone exposure via soy based food. Coefficients reflect the number of portions consumed each month according to survey response (e.g. 2-3 times a week corresponds to 10 times a month). In order to calculate each respondent's monthly (and then daily) exposure, the exposure to each type of product was summed

	Mean dose / portion mg/portion	Number of values to get the mean	*Monthly isoflavone exposure to each type of product according to survey response*				
			Never or less than once a week (coeff = 0)	*once a week (coeff = 4)*	*2-3 times a week (coeff = 10)*	*4-6 times a week (coeff = 20)*	*Every day (coeff = 28)*
1- Soy-based drinks	41.97	3	0	167.89	419.73	839.47	1,175.25
2- Soy-based steaks	52.60	7	0	210.40	526.00	1052.00	1,472.80
3- Soy-based sausages	36.38	4	0	145.54	363.86	727.72	1,018.80
4- Tofu	39.51	6	0	158.05	395.13	790.25	1,106.35
5- Biscuits/Cakes/Pancakes	33.68	4	0	134.73	336.83	673.66	943.12
6- Soy dessert cream and yogurt	27.19	8	0	108.77	271.93	543.85	761.39
7- Soy-based cheese	27.60	3	0	110.41	276.025	552.05	772.87
8- Toasted grains as appetizers	31.63	4	0	126.54	316.34	632.68	885.76
9- Tempeh	19.69	2	0	78.80	197.00	393.99	551.59
10- Flakes in breakfast mixture	18.69	2	0	74.77	186.93	373.86	523.41
11- Prepared dishes based on soy	18.10	8	0	72.40	181.00	362.00	506.80
12- Vegan chopped dish for pasta	56.98	2	0	227.92	569.81	1,139.61	1,595.46
13- Others*	13.87	16	0	55.48	138.70	277.40	388.36

*Soy raw material not taken into account for the exposure calculation

Table S10b. Data considered for the calculation of the isoflavone exposure via casual food. Coefficients reflect the number of portions consumed each month according to survey response (e.g. several times a week corresponds to 8 times a month). In order to calculate each respondent's monthly (and then daily) exposure, the exposure to each type of product was summed

Type of product	Isoflavones / portion (mg/portion)	*Monthly isoflavone exposure to each type of product according to survey response*			
		Less often or never (coeff 0)	*Once or twice a month (coeff 1.5)*	*Once a week (coeff 4)*	*Several times a week (coeff 8)*
A- Parmentier pie	15.0	0	22.5	60.0	120.0
B- minced beef / burger	10.2	0	15.3	40.8	81.6
D- Meat ball	19.5	0	29.3	78.1	156.2
E- Breaded meat	5.6	0	8.4	22.3	44.6
F- Lasagnes	2.2	0	3.3	8.7	17.4
G- Chicken Nuggets	0.2	0	0.4	1.0	1.9
H- Stuffed tomatoes	9.0	0	13.4	35.8	71.7
I- Stuffed cabbage	9.0	0	13.6	36.2	72.3
J- Brownies	10.9	0	16.4	43.6	87.3

Figure S1. Number of products by categories found on supermarket website and their content in soy, pea, plant protein as well as those being suspect. **S1A**. Meat-products, **S1B**. Breaded-meat-products, **S1C**. Delicatessen, **S1D**. Doughnuts and nuggets, **S1E**. Processed-food in can, **S1F**. Snacks, **S1G**. Ice-cream and desserts, **S1H**. Gluten-free products.

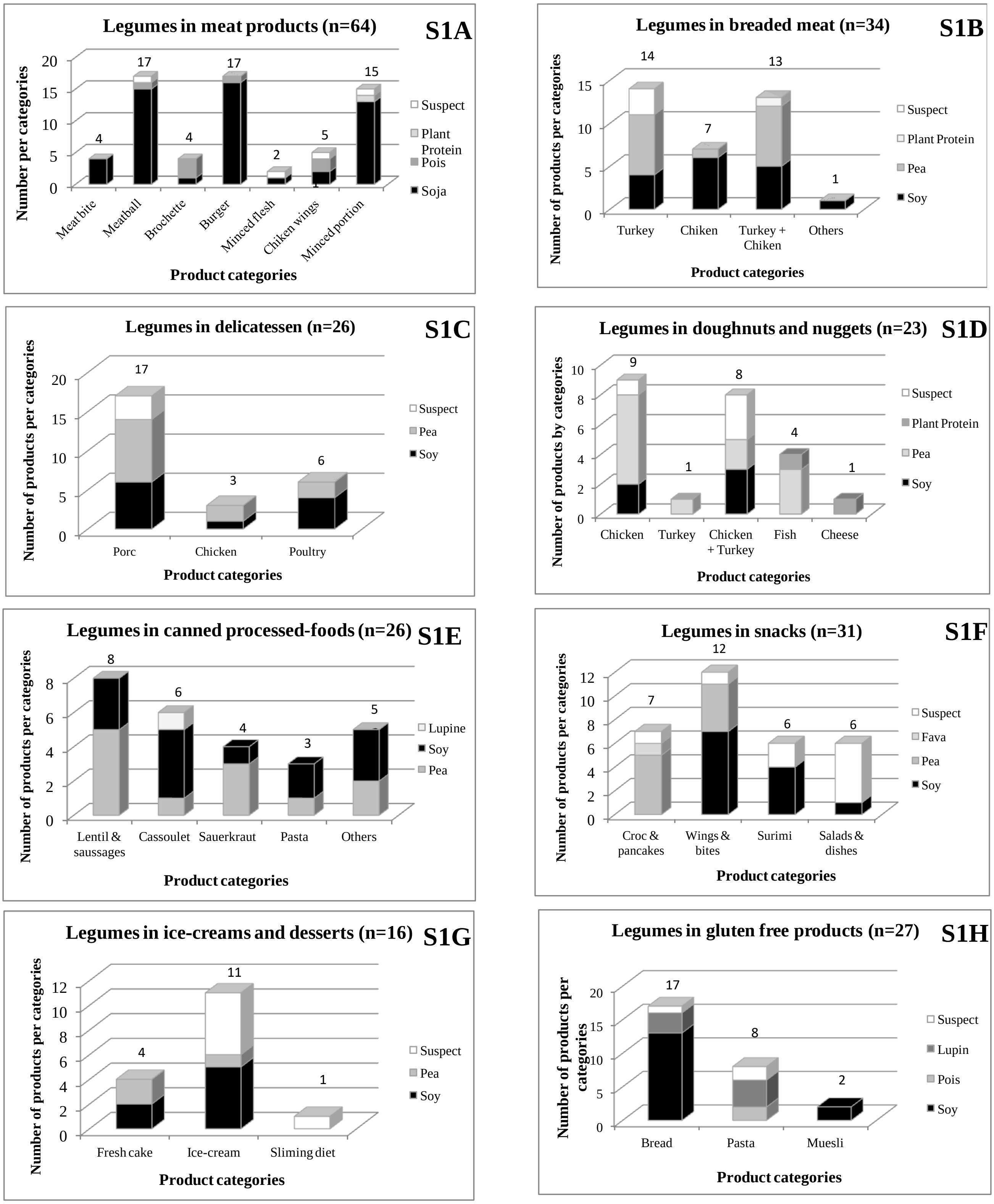

Figure S2: Characterisation of the soy-based foodstuffs offer

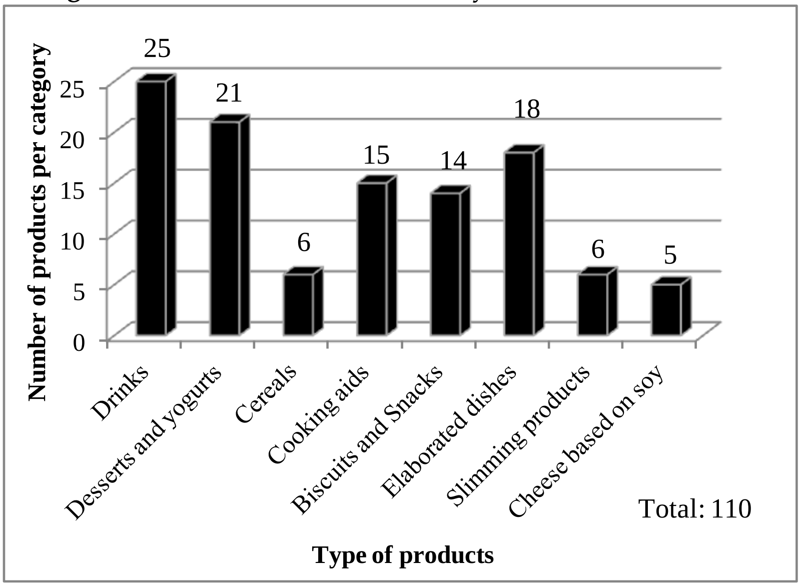

Figure S3. Proportions of each type of canned legumes in the offer of one French retailer

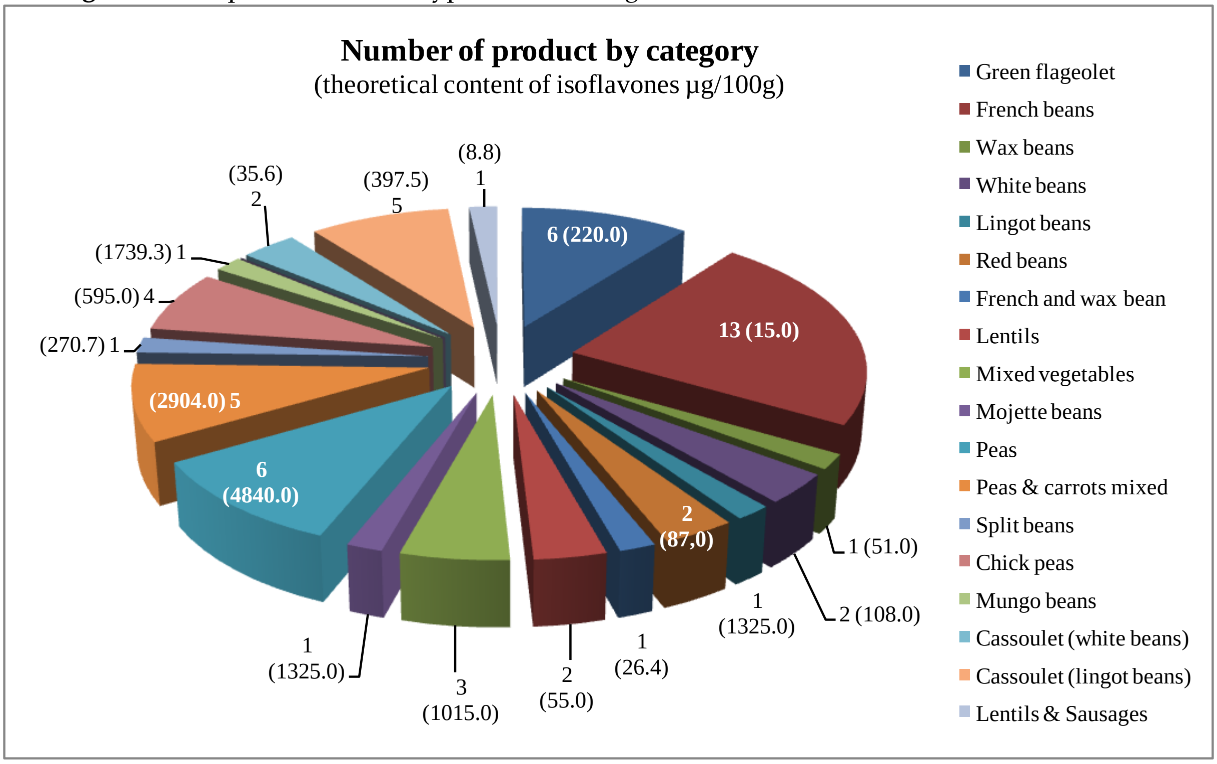

Data S1. Survey questionnaire to premenopausal women

1. This survey is for adults from 18 to 50 years-old. Is it your situation?
If yes what is your age range?

❍ 18-25 ❍ 26-30 ❍ 31-35 ❍ 36-40 ❍ 41-50

I- FOOD CONSUMPTION

2. Would you say that you pay: ❍ great attention ❍ attention ❍ low attention ❍ no attention **To your diet**

3. Are you following a specific diet?

❍ no ❍ vegetarian ❍ vegan ❍ cholesterol free ❍slimming ❍ kosher/hallal

❍ intolerant, precise____________________ ❍gluten free ❍ lactose free

❍Others, precise :______________________________________

4. Where do you mainly buy your food *(cite the two main locations)*

❍ Medium and large supermarkets (including drives) ❍ Hard-discount (Lidl, Leader Price, Aldi …)

❍ Organic shops (BioCoop - SoBio - La vie claire …) ❍ local shops (groceries, slaughters)

❍ Markets ❍ direct sales (farm, farmer drive, AMAP …)

❍ Others, precise______________________________________

5. Can you have meals in:

	Several times a week	One time a week	Less often or never
Cantinas *(from companies or universities)*?	❍	❍	❍
Fast-foods/food-trucks?	❍	❍	❍

6. Can you eat the following dishes in cantinas or in ready-made dishes *(cans, trays, frozen goods)*.
If yes at what frequency *(give an estimate)*?

	Several times a week	Once a week	Once or twice a month	Less often or never
Chopped meat pie	❍	❍	❍	❍
Minced beef /Burgers	❍	❍	❍	❍
Fish chopped steak	❍	❍	❍	❍
Meat balls	❍	❍	❍	❍
Breaded meat	❍	❍	❍	❍
Lasagnes	❍	❍	❍	❍
Chicken nuggets	❍	❍	❍	❍
Stuffed tomatoes	❍	❍	❍	❍
Stuffed Cabbages	❍	❍	❍	❍
Brownies	❍	❍	❍	❍

7. Do you pay attention to labelling looking at food composition and ingredients when you buy your food?

❍ yes for all products ❍ only on a few products : ______________________________ ◎ no

____

__Do you often read labels__

8. In this list what are the two elements that you read at first? *(prioritize them, 1 = the main)*

___ The food fact ___ The origin ___The trade mark ___ The ingredient list

___ Allergens ___ others :____

9. Soy in transformed food does it influence your choice

❍ favourably ❍ unfavourably ❍ No impact

II- SOY COMSUMPTION

10. Do you consume Soy-based products (sauces, juice, creams, tofu etc.)**?**

❍Yes ❍ No

__If not :__

11'. Did you consume these products in the past?

❍ Yes ❍ No

12'. __If yes__, why did you stop?____

____

__If yes:__

11. For how long do you consume soy?

❍ less than a year ❍ from 1 to 3 years ❍ from 3 to 10 years ❍ more than 10 years

12. What types of products do you eat and how often?

	Every day	4 to 6 times a week	2 to 3 times a week	Once a week	Less often or never
Soy based juice	❍	❍	❍	❍	❍
Soy based steak	❍	❍	❍	❍	❍
Soy based sausages	❍	❍	❍	❍	❍
Tofu	❍	❍	❍	❍	❍
Cookies, pancakes…	❍	❍	❍	❍	❍
Yogurts or dessert cream	❍	❍	❍	❍	❍
Cheese	❍	❍	❍	❍	❍
Toasted soy grain as appetizer	❍	❍	❍	❍	❍
Tempeh	❍	❍	❍	❍	❍
Flakes/Soy based cereals	❍	❍	❍	❍	❍
Prepared dish based on soy	❍	❍	❍	❍	❍
Vegan minced dish	❍	❍	❍	❍	❍
Other:________________	❍	❍	❍	❍	❍

13. Why do you consume these products?

❍ I enjoy the taste ❍ It's better for the environment ❍ It's cheaper than animal proteins ❍ It's better for health ❍ It's easy to cook

❍ To replace animal proteins ❍ Other, precise ____________________

14. Do you have favourite trademarks *(2 or 3 maximum)* **?**

❍ Yes, precise ____________________ ❍ No

15. Except you, who consume soya at yours? *(several answer possible)*

❍ Nobody ❍ cohabitants ❍ child less than 3 ❍ child over 3 ❍ adolescent

❍ Other, precise ____________________

III- Soybean Perception – Consumer consciousness

16. As food, what is (are) the word(s) that you would spontaneously associate to soy?

____

17 For you is soy associated to a balanced diet?

❍ Yes ❍ No ❍ I don't Know

18. Were you already advised to eat soy-based food?

❍ Yes ❍ No

19. If yes, was it: *(several answers are possible)*

❍ a medic ❍ a nutritionist ❍ a dietician ❍ a relative ❍ medias

For what raison(s)? ____________________

20. Do you think that regular soy consumption can have health effects:

❍ essentially positive ❍ neutral ❍ essentially negative ❍ I don't know

If positive or negative. **Can you precise:** ____________________

____

21. More precisely do you think that regular soy consumption can have effects on:

	Positive	No effect	Negative	No clue
- Allergies?	❍	❍	❍	❍
- Cancer risks?	❍	❍	❍	❍
- Sport achievements?	❍	❍	❍	❍
- Infants?	❍	❍	❍	❍
- Cardiovascular diseases?	❍	❍	❍	❍
- Thyroid?	❍	❍	❍	❍
- Brain ageing?	❍	❍	❍	❍
- Hormonal cycles?	❍	❍	❍	❍
- Diabetes?	❍	❍	❍	❍
- Fertility?	❍	❍	❍	❍
- Weight?	❍	❍	❍	❍

22. Except Breast-feeding do you think that it is better to feed infants with milk from:

❍ animals ❍ plants ❍ no clue

23. Do you think that you are informed enough about soybean effects on health?

❍ Yes ❍ No ❍ No clue

24. Did you ever eared about:

- soy isoflavones?	❍ Yes	❍ No
- phytoestrogens?	❍ Yes	❍ No

If Yes, can you tell us more: ______

Let's end by a few questions on your profile…

IV- PROFILE

25. How many persons are leaving at your house? (including you) ____

26. Do you have children? ❍ Yes ❍ No

27. What is the rank of your monthly incomes?

❍ < 1500 € ❍ [1500 ; 2500[€ ❍ [2500 ; 4000[€ ❍ [4000 ; 6000[€ ❍ > 6000 €

28. What is your actual job? ______

(For people without a job ask: what is the area in which you are looking for a job? For students in what area are you studying?)

29. How often are you doing sports?

❍ every days ❍ every weeks ❍ several times a month ❍ less often ❍ never

30. Are you following a hormonal treatment?

❍ Yes, precise : ______ ◎ No

31. Do you want to add something to this inquiry?

______

______

Many Thanks !
